# Supplementary figures and images for: CryoEM structures of the human CLC-2 voltage-gated chloride channel reveal a ball-and-chain gating mechanism
Source: eLife. 2024 Feb 12;12:RP90648. doi: 10.7554/eLife.90648 (PMC10942593; doi:10.7554/eLife.90648)

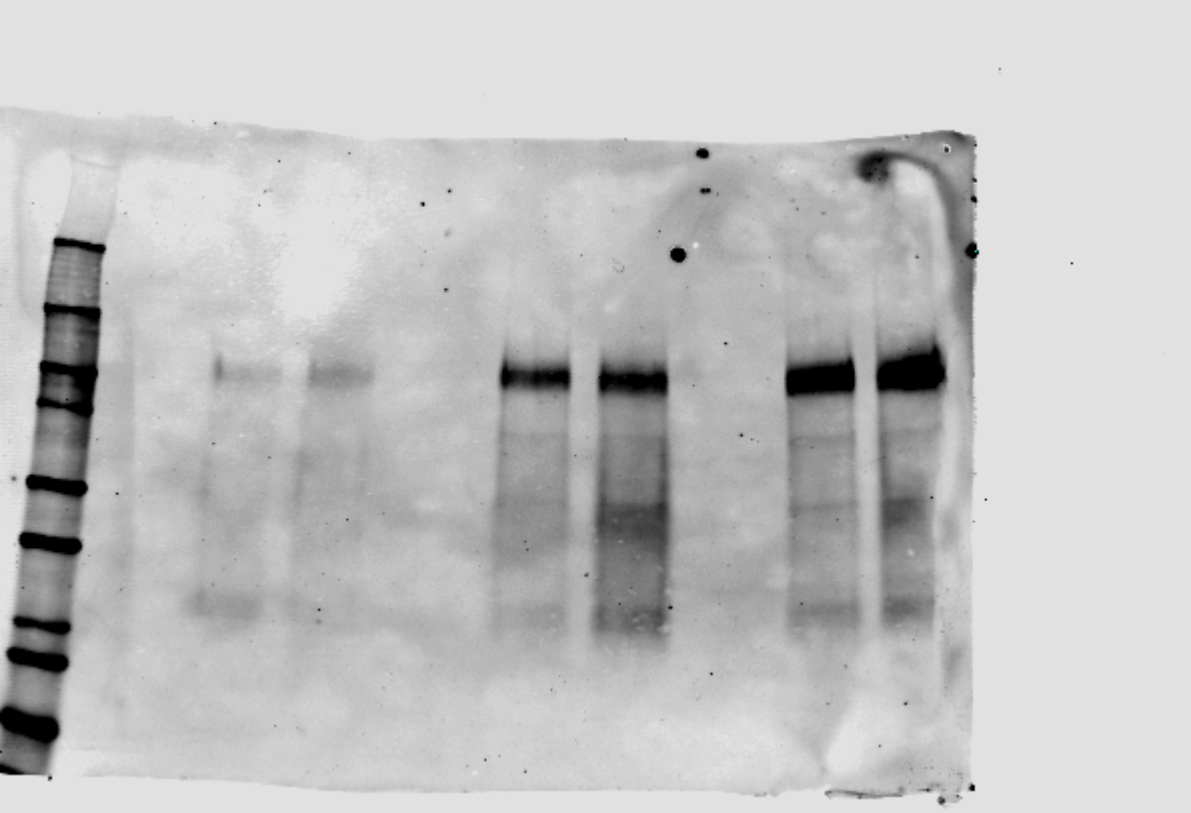

Supplement: Figure 6—source data 1. [file elife-90648-fig6-data1.zip › Figure 6 source data 1.png]

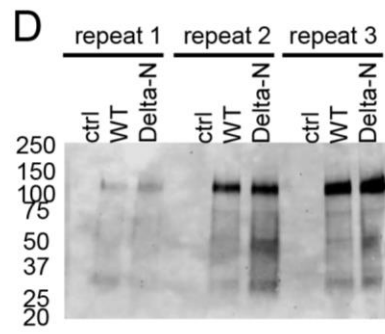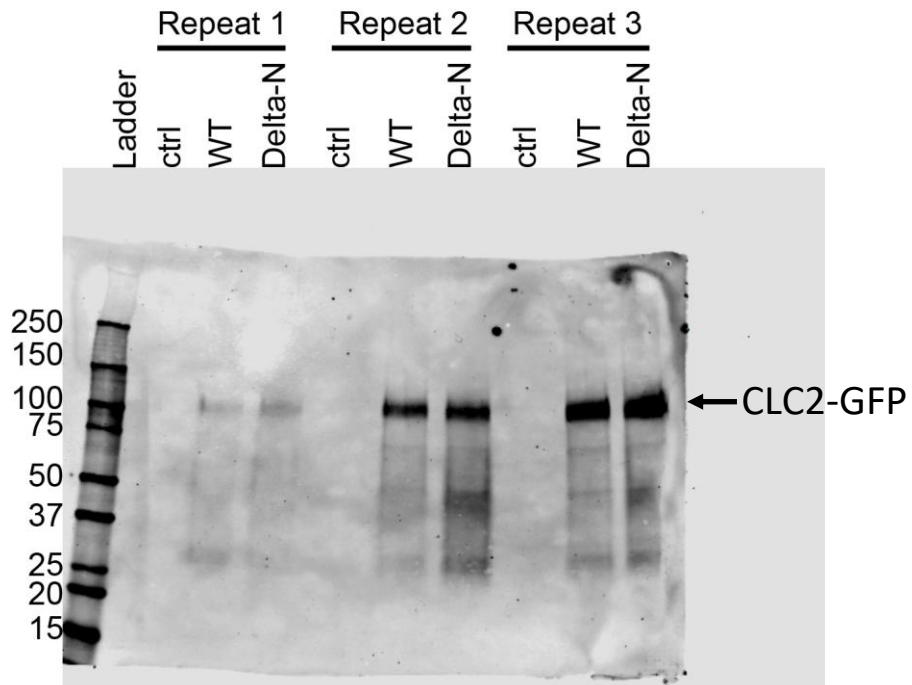

Supplement: Figure 6—source data 2. [file elife-90648-fig6-data2.zip › Figure 6 source data 2.pdf]
