# Supplementary material for: CryoEM structures of the human CLC-2 voltage-gated chloride channel reveal a ball-and-chain gating mechanism
Source: eLife. 2024 Feb 12;12:RP90648. doi: 10.7554/eLife.90648 (PMC10942593; doi:10.7554/eLife.90648)
Supplement: Supplementary file 1. [file elife-90648-supp1.docx]

| **Data collection** | **CLC2-TM** | **CLC2-CTDsym** | **CLC2-CTDasym** | **CLC2-TM-AK42** | **CLC2-AK42-CTD-only** |
| --- | --- | --- | --- | --- | --- |
| Microscope | Titan Krios (Thermo Fisher Scientific) | | | | |
| Voltage (keV) | 300 | | | | |
| Electron exposure (e^-^/Å^2^) | 50 | | | | |
| Detector | Falcon 4 (Thermo Fisher Scientific) | | | | |
| Magnification | ×130,000 | | | | |
| Defocus range (μm) | -1.0~-2.0 | | | | |
| Pixel size (Å/pix) | 0.946 | | | | |
| Number of movies | 14,198 | | | 14,300 | |
| Symmetry | C1 | | | | |
| Picked particles | 4,999,780 | | | 5,214,695 | |
| Final particles | 2,415,222 | 56,580 | 39,272 | 2,391,813 | 66,251 |
| Map resolution (Å) | 2.47 | 2.75 | 2.76 | 2.74 | 4.03 |
| FSC threshold | 0.143 | | | | |
| **Model refinement** | | | | | |
| Non-hydrogen atoms | 7,292 | 9,734 | 9,387 | 7,168 | 2,158 |
| **R.m.s. deviations for ideal** | | | | | |
| Bond lengths (Å) | 0.013 | 0.012 | 0.012 | 0.067 | 0.012 |
| Bond angles (°) | 1.93 | 1.93 | 1.92 | 2.02 | 2.03 |
| **Validation** | | | | | |
| Clashscore | 0.13 | 0.10 | 0.05 | 0.07 | 0.91 |
| Rotamers (%) | 0.92 | 1.36 | 1.00 | 1.74 | 2.10 |
| **Ramachandran plot** | | | | | |
| Favored (%) | 97.39 | 96.75 | 97.30 | 97.15 | 90.23 |
| Outlier (%) | 0.11 | 0.08 | 0.08 | 0.11 | 0.38 |
